# Fermented cottonseed meal improves production performance, immune function and intestinal microecological environment of laying hens and its nutritional properties

**DOI:** 10.3389/fvets.2025.1622229

**Published:** 2025-09-22

**Authors:** Mingyang Xu, Jia Li, Wei Wang, Tongguo Gao, Caixuan Zhang, Zhimin Hao, Cheng Zhou

**Affiliations:** ^1^College of Life Sciences, Hebei Agricultural University, Baoding, Hebei, China; ^2^School of Light Industry Science and Engineering, Beijing Technology and Business University, Beijing, China; ^3^Central Laboratory, Affiliated Hospital, Hebei University, Baoding, Hebei, China

**Keywords:** fermented cottonseed meal, feed, laying hens, nutrition, intestinal health, metabolomic profile

## Abstract

**Introduction:**

Global population growth and consumption upgrades have led to an increase in demand for animal feed protein sources, which has prompted an urgent need to find economical alternative protein sources. In this study, the potential of partially replacing traditional protein source soybean meal (SBM) with fermented cottonseed meal (FCSM) in practical applications were evaluated.

**Methods:**

The 180 24-week-old Hyland Brown laying hens were randomly divided into a control group (CON, fed SBM) and an experimental group (FCSM, fed FCSM). The effects of FCSM on the production performance, immune system, intestinal morphology and microbiota composition of laying hens were investigated. Furthermore, the enrichment of FCSM for characteristic nutrient metabolites and key pathways was further explored.

**Results:**

The feed-to-egg ratio and egg quality indexes (eggshell thickness, eggshell strength, albumen height and Haugh units) of laying hens in the FCSM group were significantly better than those of the CON group (*p* < 0.05). Additionally, splenic index, immunoglobulins (IgG, IgM and IgA) and albumin/globulin ratio were stable in the FCSM group. FCSM significantly increased the species richness of the gut flora as well as the beneficial bacteria such as *Rikenellace_ae_RC9_gut_*group and *Romboutsia* (*p* < 0.05). Non-targeted metabolomics analyses indicated that microbial solidstate fermentation technology increased the levels of nutrients in CSM, such as L-glutamine, ornithine, citrulline, and D-galactose.

**Conclusion:**

FCSM promoted the absorption of nutrients and intestinal health in laying hens by regulating the intestinal flora, demonstrating superior efficacy over BSM in overall production performance, immune function, and intestinal morphology. These findings provides a theoretical basis for the resource reuse in CSM and the precise nutrition of laying hens.

## 1 Introduction

Cottonseed meal (CSM), as a potential alternative raw material, has significant advantages in terms of crude protein content (222–562 g/kg) and amino acid composition (1). However, the unbalanced amino acid profile and the presence of anti-nutritional factors such as free gossypol (FG) limit its application in poultry diet (1). Studies have shown that the range of FG content in CSM is 200–5,300 mg/kg, while the upper limit of safe concentration of FG in laying hens’ diets is only 40 mg/kg (2). FG, as a polyphenolic compound, not only binds to the *ε*-amino group of lysine and reduces its bioavailability (3, 4), but also inhibits the activities of pepsin and trypsin in the gastrointestinal tract, thereby affecting protein digestion and absorption (5, 6). In addition, FG has significant toxic effects on the growth performance and physiological metabolism of poultry, including growth inhibition, liver injury and lipid metabolism disorders (7). It is worth noting that there were significant differences in the tolerance of FG in poultry at different growth stages, suggesting that the application of CSM needs to be optimized according to the physiological characteristics of poultry. Currently, although several physical and chemical methods have been used to reduce the FG content in CSM, these methods often lead to problems such as loss of active vitamins, decrease in protein quality, and decrease in feed palatability (8, 9). Therefore, the development of efficient, environmentally friendly and economical FG detoxification technologies has become the focus of current research on CSM resource utilization. This will not only help to alleviate the contradiction between the supply and demand of traditional protein feed resources, but also provide a new solution for the sustainable development of the poultry industry.

In recent years, the application of microbial solid-state fermentation technology in improving the nutritional value of CSM has attracted much attention (10). This technology can effectively degrade anti-nutritional factors such as FG through biochemical reactions during microbial metabolism, and at the same time improve the bioactivity and digestibility of feed (11, 12). Studies demonstrated that solid-state fermentation of CSM using specific microorganisms, such as *Bacillus subtilis* and *Saccharomyces cerevisiae*, significantly reduced the FG content, thereby improving its safety and nutritional value as a feed ingredient (13, 14). Wang et al. further demonstrated that *B. subtilis* was able to significantly improve the health of the animals by regulating the balance of the intestinal flora and enhancing the intestinal barrier function (15). In addition, our previous studies showed that *B. subtilis* fermentation could effectively degrade the antinutritional factors in CSM and increase its crude protein, small peptide content and *in vitro* protein digestibility (16). Fermented feeds not only degrade antinutritional factors, but also enrich functional ingredients such as lactic acid bacteria (LAB), lactic acid and other organic acids, which play an important role in promoting intestinal health and improving animal production performance (17, 18). Recent years, fermented feeds have received much attention due to their unique nutritional properties, digestibility, palatability and safety. Beneficial microorganisms, enzymes and metabolites produced during the fermentation process have been shown to significantly improve performance, immunity and gastrointestinal ecological regulation in broilers (19). However, despite the significant progress in broiler research, studies on laying hens are still relatively limited, especially the effect mechanisms of CSM fermented by *B. subtilis* on immune function, intestinal flora and production performance in laying hens remain unclear.

In our previous work, fermented CSM (FCSM) with significantly reduced FG content and improved nutritional quality was obtained by anaerobic solid-state fermentation with *B. subtilis* M-15 (16). To evaluate the potential application of FCSM in laying hens diets, we further investigated the effects of FCSM on laying hens production performance, egg quality, immune function, intestinal morphology and microbial community. Furthermore, we used non-targeted metabolomics to explore the characteristic metabolites and key metabolic pathways of FCSM and elucidated the mechanism of its enhanced nutritional value. This study provides a new approach to optimize feed formulation for laying hens and offers a viable solution to the protein feed shortage problem.

## 2 Materials and methods

### 2.1 Experimental design and feeding management

This study involving live animals met the guidelines of the Institutional Animal Care and Use Committee (IACUC). The animal use protocol was approved by the Institutional Animal Care and Use Committee of Hebei Agricultural University. All data were collected by adhering strictly to the requirements of this Committee. All test methods are designed and operated according to ARRIVE guidelines. Utmost efforts were taken to minimize the number of animals used with little or no suffering.

One hundred and eighty 24-week-old Hyland Brown laying hens (purchased from a farm in Baoding) with similar body weights were selected. Subsequently, they were randomly divided into 2 treatment groups with 6 replicates each (15 birds/replicate). The basal diets (Table 1) were formulated according to the National Research Council (NRC) (1994) Nutrient requirements of poultry. Soybean meal (SBM) and FCSM were used as protein sources in the control (CON) and experimental (FCSM) groups, respectively. The experiment was conducted for a period of 45 days, which coincided with the transition of laying hens from the early stage of egg production to the stable stage of egg production. The first 5 days were an acclimatization period in which the groups were fed regular basal diets, and 6–25 days were fed diets supplemented with SBM or FCSM, respectively. On days 26–45, the feed formulation was further adjusted according to Table 1. Laying hens were fed twice a day, once at 8:00 and once at 14:00, with free access to water. The feeding experiment was conducted at the experimental base of Hebei Agricultural University, and the management was carried out according to the conventional management mode of chicken farms (14–20 °C, humidity 45–60%, 16 h light/day). Cages were made of nickel-plated wire cages with flue heating and thermal insulation. The laying hens were vaccinated according to the routine immunization program. Egg collection was carried out three times a day at 10:00, 15:00 and 18:00 and egg production was counted.

**Table 1:** Formulation and nutrient composition of the experimental diets.

Items	FCSM		CON	
	Applicable phase			
	6–25 d	26–45 d	6–25 d	26–45 d
Formulation composition (%)				
Yellow corn	50	50	50	50
Maize	–	10	–	10
Soybean cake powder	5	–	25	17
Broken rice	5	5	5	5
Bran	10	12	10	12
Fishmeal	7.5	4.5	7.5	4.5
Meat powder	1	–	1	–
Shell powder	1	1	1	1
NaCl	0.5	0.5	0.5	0.5
FCSM	20	17	–	–
Total	100	100	100	100
Nutrient composition				
Metabolizable energy (MJ/kg)	12.24	12.89	12.54	13.26
Crude protein (%)	19.4	16.8	20	17.5
Crude fiber (%)	4.05	4.1	3.9	4.1
Calcium (%)	1.0	0.5	1.1	0.5
Available phosphorus (%)	0.5	0.5	0.5	0.5
FG (mg/kg)	11.64	9.89	–	–

FCSM, fermented cottonseed meal; CSM, cottonseed meal; FG, free gossypol; d, days. “–” indicates that the nutrient is not contained.

### 2.2 Production performance and egg quality

The egg production (EP), egg laying rate (ELR), average egg weight (AEW), death rate during the egg laying (DR) and feed-to-egg ratio (FER) were recorded and calculated during feeding.

The albumen height (AH), yolk color (YC), and Haugh units (HU) were measured using an egg quality analyzer (EMT-7300, Bulader, Beijing, China). The Eggshell thickness (EST) and strength (ESS) were tested by using an eggshell thickness tester (ETG-1601A, Robotmation, Tokyo, Japan) and an eggshell strength tester (EFG-0503, Robotmation, Tokyo, Japan), respectively. Eggshell ratio is the ratio of eggshell weight to total egg weight.

In order to evaluate the effect of feeding FCSM on the palatability of eggs produced by laying hens, a sensory evaluation panel consisting of 20 professionals was set up and a double-blind test method was used for sensory evaluation. A 9-point scale was used to score the fishy odor (FO) of raw eggs and the aroma (AR), aftertaste (AF) and overall acceptability (OA) of cooked eggs. A score of 1 indicates that the attribute is absent or strongly disliked, while a score of 9 indicates that the attribute is very strong or strongly liked.

### 2.3 Immune organ index and serum biochemical tests

After 12 h of fasting at the end of feeding, 2 laying hens with similar body weight were randomly selected from each replicate. 10 mL of blood was taken from the laying hens through bronchial veins, allowed to stand for 30 min, and then centrifuged at 3000 x g for 20 min at 4 °C to obtain serum samples (Thermo Scientific Sorvall Legend Micro 21R, Thermo Scientific, United States). Serum samples were stored at −80 °C for subsequent analysis.

After slaughter and cleaning, the spleen was isolated, followed by removal of fat and connective tissues and quickly weighed. The immune organ index was calculated by the following formula: weight of immune organ/weight of the whole laying hen (g/kg). Immunoglobulins IgG, IgA, and IgM in serum were detected by ELISA kits (Nanjing Jianjian Bioengineering Institute, Nanjing, China), and the detection process was performed by double antibody sandwich method. Total protein (TP), albumin (ALB) and globulin (GLB) were determined by an automatic biochemical analyzer (BS-240 Vet, Mindray Animal, Shenzhen, China).

### 2.4 Pathological structure of spleen and intestinal morphology analysis

Referring to the method of Wang et al. (20), tissue samples of the spleen, duodenum, jejunum and ileum were fixed in 4% paraformaldehyde, embedded in paraffin and sectioned. Tissue sections were stained with hematoxylin and eosin (H&E) and photographed under the microscope for observation. Then, villus height (VH) and crypt depth (CD) were measured using ImageJ software (National Institutes of Health, Bethesda, Maryland), and the ratio of VH to CD was calculated.

### 2.5 Gut flora analysis

Total DNA (DP302, TianGen, Beijing, China) was extracted from the cecum using a fecal DNA kit, and the quality of the extracted genomic DNA was measured by 1% agarose gel electrophoresis. The concentration and purity of the DNA were determined by NanoDrop2000 (Thermo Scientific, United States). The sequencing was performed on the Illumina NextSeq 2000 platform by Shanghai Majorbio Technology Co.

Based on the OTUs/ASVs information, rarefaction curves and alpha diversity indices including observed OTUs/ASVs, Chao1 richness, Shannon index, and Good’s coverage were calculated with Mothur v1.30.1. Differences in microbial community structure between groups were assessed using principal coordinates analysis (PCoA) based on Bray-Curtis distance and PERMANOVA tests. The linear discriminant analysis (LDA) effect size (LEfSe) was performed to identify the significantly abundant taxa (phylum to genus) of bacteria among the different groups (LDA score > 2, *p* < 0.05).

### 2.6 Untargeted metabolomics analysis

The *Bacillus* culture were inoculated with 1 × 10^9^ CFU/kg in unsterilized SSF medium (CSM 80.0%, corn meal 17.0%, (NH_4_)_2_SO_4_ 3.0%). The inoculated medium was mixed with an equal volume of water and subsequently packed into 6 kg capacity buckets, finally compacted and sealed. Fermentation was carried out at room temperature for 14 days. Each group had six parallel samples. Samples were taken before and after fermentation, respectively.

50 mg of pre- and post-fermentation samples were mixed and ground with 400 μL of extraction solution (methanol: water = 4:1 (v:v)) containing the internal standard (L-2-chlorophenylalanine). Then the mixture was extracted by low-temperature sonication for 30 min (5 °C, 40 kHz), left to stand for 30 min (−20 °C), centrifuged for 15 min (4 °C, 13,000 g), and finally the extraction supernatant was collected. Sequencing was performed by Shanghai Majorbio Co., Ltd. The data were analyzed through the free Majorbio cloud platform.^1^ Screening for differential metabolites was performed as follows, based on orthogonal partial least squares discriminant analysis (OPLS-DA), using *t*-tests to assess whether statistical differences existed between groups, and selecting metabolites with VIP > 1 and *p* < 0.05 as potential differential metabolites.

### 2.7 Statistical analysis

Data are expressed as mean ± standard deviation (SEM). IBM SPSS Statistics 21.0 software (SPSS Inc., United States) was employed to conduct one-way analysis of variance (ANOVA) and a *T*-test. Origin 2018 for data processing and plotting. *p* < 0.05 was considered statistically significant and 0.05 < *p* < 0.1 was considered to indicate a trend. A correlation between two nodes was considered to be statistically robust if Spearman’s correlation coefficient was over 0.6 or less than −0.6, and the *p*-value was less than 0.01.

## 3 Results

### 3.1 Production performance and egg quality

In this study, the effect of FCSM replacing SBM on the production performance of laying hens was evaluated by comparing the production performance indexes of FCSM and CON groups. As shown in Table 2, EP, ELR, AEW and DR of FCSM group were not significantly different from CON group (*p* > 0.05). Nevertheless, FER of FCSM group was significantly better than CON group (*p* < 0.05).

**Table 2:** Production performance indicators of laying hens.

Items	CON	FCSM
EP (g/d)	52.50 ± 1.10^a^	53.80 ± 1.11^a^
ELR (%)	87.90 ± 1.21^a^	88.60 ± 1.17^a^
AEW (g/piece)	59.90 ± 0.09^a^	59.50 ± 0.08^a^
DR (%)	10.00 ± 0.67^a^	8.89 ± 0.89^a^
FER	1.91 ± 0.12^b^	2.02 ± 0.11^a^

d, days; EP, egg production; ELR, egg laying rate; AEW, average egg weight; DR, death rate during egg laying; FER, feed-to-egg ratio; CON, control group; FCSM, experimental group. Different lowercase letters indicate significant differences.

The effect of FCSM on egg quality was further analyzed (Table 3). The results demonstrated that the Eggshell ratio and YC indexes in the FCSM group were slightly lower than those in the CON group, whereas the EST, ESS, AH, and HU were significantly better than those in the CON group (*p* < 0.05). Notably, the EST of FCSM group was more than 0.32 mm, which met the standard for high-quality eggs (21).

**Table 3:** Egg quality indicators.

Items	CON	FCSM
Eggshell ratio (%)	8.91 ± 0.42^a^	8.86 ± 0.25^a^
EST (mm)	0.321 ± 0.0026^b^	0.347 ± 0.0012^a^
ESS (kgf)	3.69 ± 0.011^b^	3.88 ± 0.015^a^
AH (mm)	5.77 ± 0.067^b^	5.91 ± 0.052^a^
YC	6.57 ± 0.032^a^	5.98 ± 0.018^b^
HU	89.22 ± 0.23^b^	91.82 ± 0.16^a^

EST, eggshell thickness; ESS, eggshell strength; AH, albumen height; YC, yolk color; HU, Haugh units; CON, control group; FCSM, experimental group. Different lowercase letters indicate significant differences.

The results of Supplementary Table S1 showed that there was no statistically significant difference in the taste of eggs produced by the two groups of laying hens. This indicated that eggs from FCSM-fed laying hens did not have undesirable flavors. However, it is noteworthy that the frequency of blood-spotted eggs was significantly reduced after feeding FCSM to the laying hens. The above suggested that FCSM may play an important role in improving the quality of egg production.

### 3.2 Histopathological sections of immune organs and serum immunity indicators

**Figure 1:**
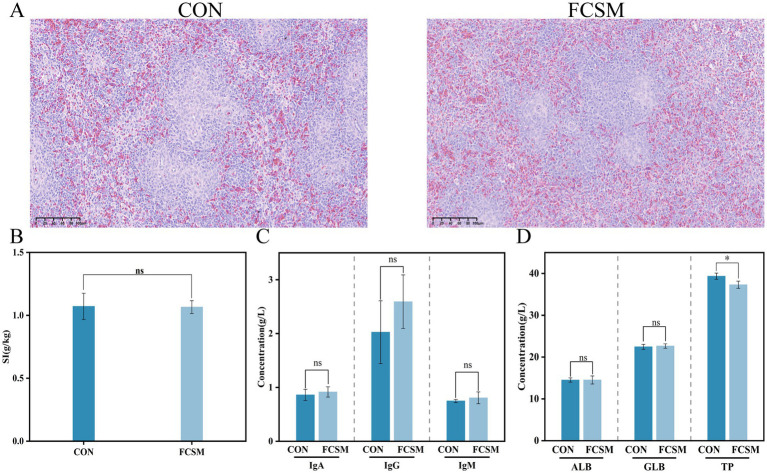
**(A)** Pathological morphology of spleen in laying hens fed SBM and FCSM basal diets (scale bar 100 μm). **(B)** Splenic index. **(C)** Serum immunoglobulin concentration. **(D)** Serum protein index. “ns” indicates no statistical significance. “***” indicates *p* < 0.001. “**” indicates *p* < 0.01. “*” indicates *p* < 0.05.

Histopathological analysis of the spleen of laying hens was performed by tissue sectioning and HE staining. The results showed (Figure 1A) that the spleen tissue of laying hens in the FCSM group did not show obvious pathological damage compared with that of the CON group, indicating that FCSM did not have obvious adverse effects on the tissue structure of the spleen. In addition, the results of the spleen index showed that there was no statistically significant (*p* > 0.05) difference between the CON and the FCSM groups (Figure 1B). This further confirmed that FCSM had no significant effect on the development and function of the spleen in laying hens.

The effects of FCSM as a feed protein source on the immune system of laying hens were evaluated by measuring serum immune factors and serum TP levels (Figures 1C,D). The levels of IgG, IgM, IgA, ALB and GLB in the FCSM group were not significantly different from those in the CON group (*p* > 0.05), but the levels of TP were significantly lower than those in the CON group (*p* < 0.05). This demonstrated that FCSM had no negative effect on the immune system of laying hens and did not cause a metabolic burden. This result strongly supported the use of FCSM as a safe and effective feed protein source.

### 3.3 Intestinal morphology

**Figure 2:**
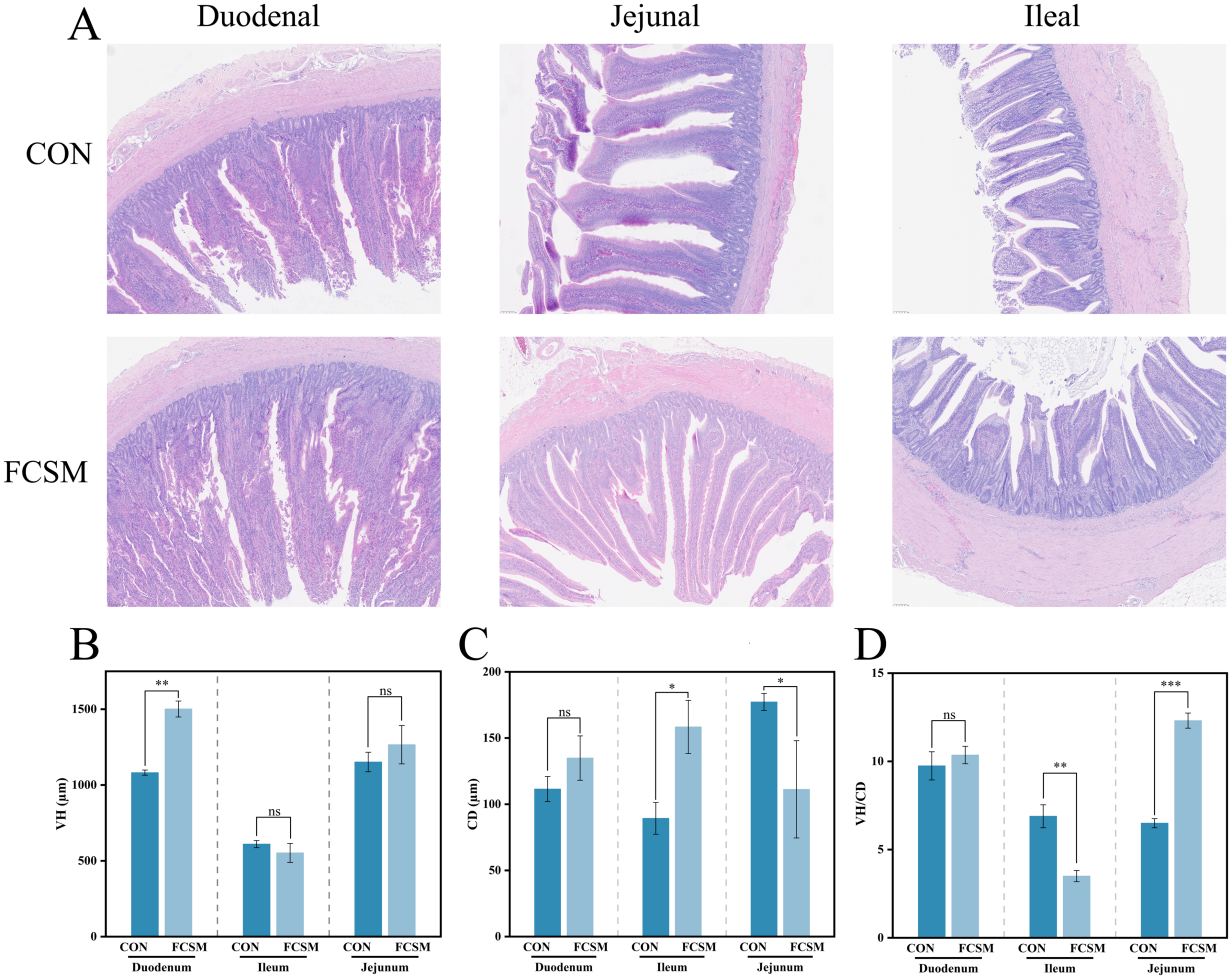
**(A)** Morphology of the duodenum, jejunum and ileum of laying hens fed the basal diets of SBM and FCSM (scale bar: 100 μm). **(B)** Velvet height (VH) of the duodenum, jejunum and ileum of laying hens fed the basal diets of SBM and FCSM. **(C)** Crypt depth (CD) of the duodenum, jejunum and ileum of laying hens fed SBM and FCSM. **(D)** Velvet height/crypt depth (VH/CD). “ns” indicates no statistical significance. “***” indicates *p* < 0.001. “**” indicates *p* < 0.01. “*” indicates *p* < 0.05.

Nutrient absorption in poultry is closely related to intestinal villus morphology. Therefore, the present study further observed the effect of FCSM on the morphology of duodenum, jejunum and ileum in laying hens (Figure 2A). The experimental results showed that FCSM significantly increased the VH of duodenum but decreased the CD of jejunum (Figures 2B,C) in laying hens. The VH/CD ratios of jejunum and ileum exhibited different changes (Figure 2D) (*p* < 0.05). These results suggested that FCSM has a significant modulatory effect on the intestinal morphology of laying hens and may promote nutrient absorption by improving intestinal structure.

### 3.4 Intestinal flora

**Figure 3:**
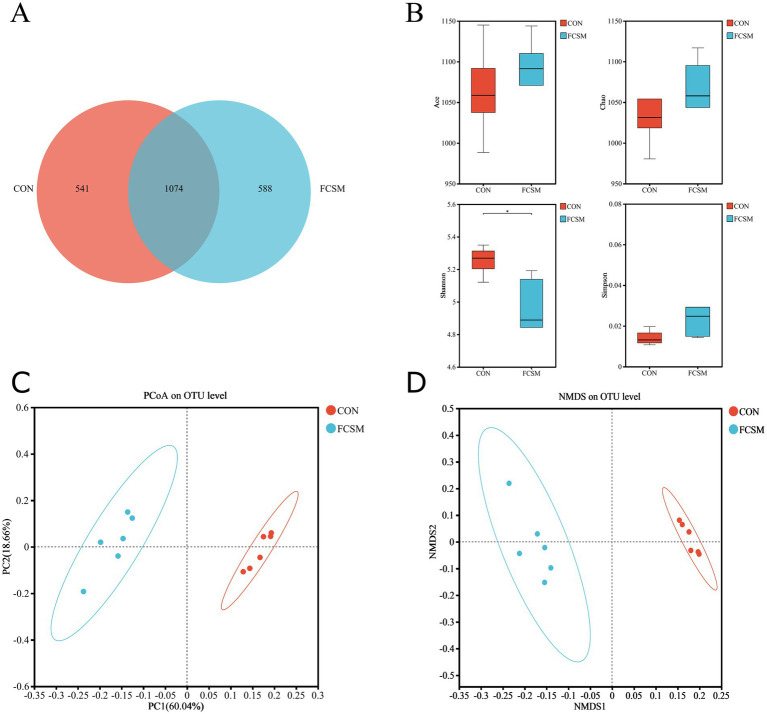
Effects of fermented cotton meal (FCSM) on cecum microbiota diversity and composition in laying hens. **(A)** Venn diagram. **(B)** ACE diversity, Chao1 diversity, Shannon diversity, and Simpson diversity in CON and FCSM groups. **(C)** PCoA analysis in CON and FCSM groups. **(D)** NMDS analysis in CON and FCSM groups.

To investigate the impact of FCSM on the cecal microflora of laying hens, 16S rRNA high-throughput sequencing was employed to analyze the compositional structure of the intestinal flora in the two groups. The total number of OTUs can reflect the diversity of the intestinal flora. As shown in Figure 3A, there were 541 and 588 independent OTUs in CON and FCSM, respectively. Larger Ace and Chao1 indices represent higher colony species richness, and larger Shannon index values represent higher colony diversity; conversely, higher Simpson’s index values represent lower colony diversity. It can be observed through Figure 3B that the species richness of FCSM was increased while the colony diversity was decreased compared to the CON group. Meanwhile, NMDS and PCoA (Figures 3C,D) showed that there was a significant difference in the intestinal flora structure between the CON and FCSM groups.

**Figure 4:**
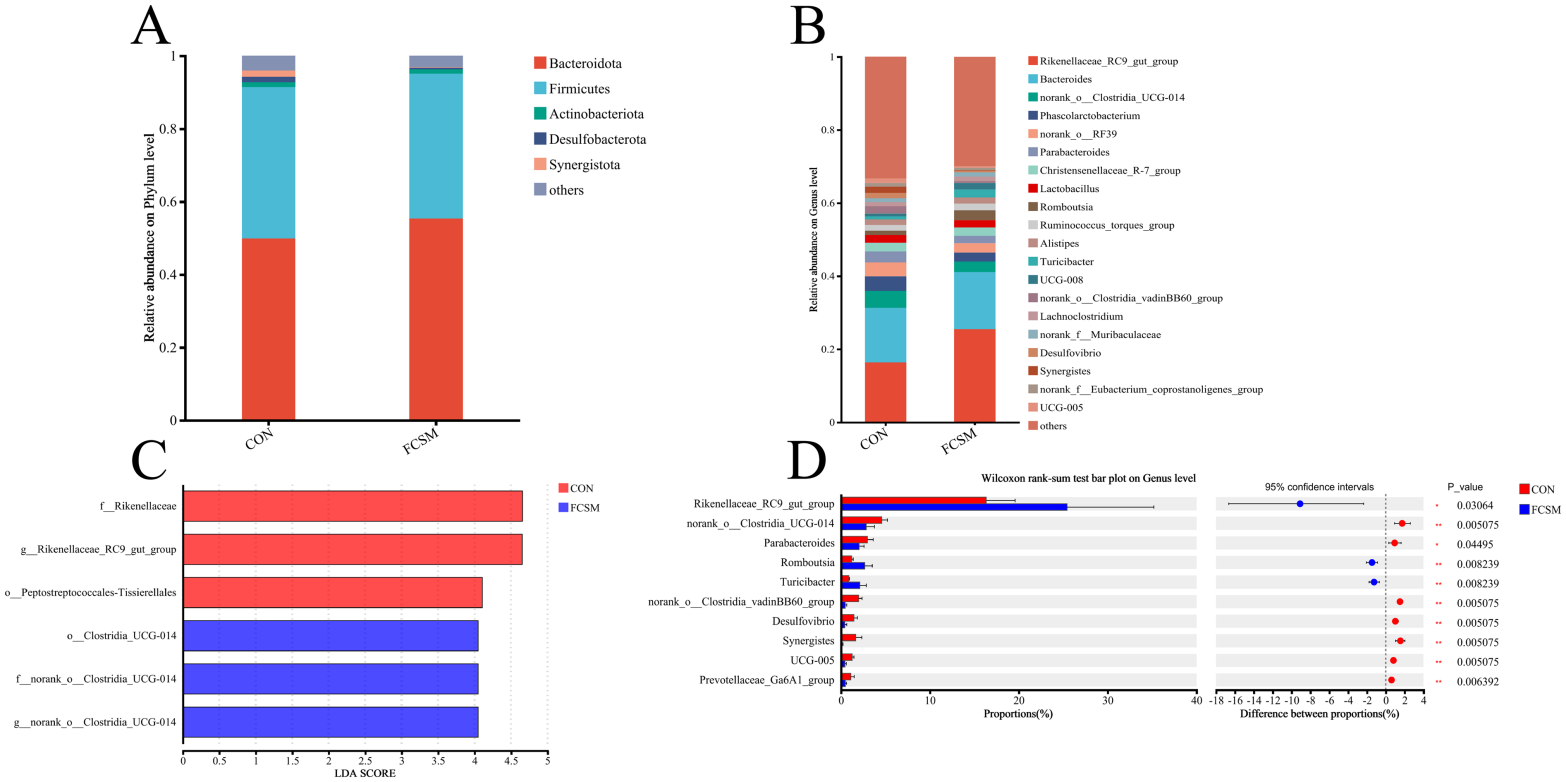
Effects of fermented cotton meal (FCSM) on the diversity and composition of cecum microbiota in laying hens. **(A)** Microbial composition at the phylum level. **(B)** Microbial composition at the genus level. **(C)** LEfSe analysis (LDA scores >3.5). **(D)** Differential analysis of Intestinal flora in laying hens between CON and FCSM groups using the Wilcoxon rank-sum test.

Feeding FCSM significantly altered the structure and function of the gut flora. The relative abundance of *Bacteroidetes* was significantly increased at the phylum level (Figure 4A), while the abundance of *Rikenellaceae_RC9_gut_group* and *Romboutsia* was significantly increased at the genus level (Figure 4B). A significant increase in the abundance of *Rikenellaceae_RC9_gut_group*, *Romboutsia*, and *Turicibacter* in the FCSM is shown in Figure 4D. The significant effect of feeding FCSM on the intestinal flora of laying hens was further verified using LEfSe analysis (LDA > 3.5) (Figure 4C). The results showed that *o_Clostridia_UCG-014* (from phylum to genus) *f_norank_o_Clostridia_UCG-014* and *g_norank_o_Clostridia_UCG-014* were significantly enriched in the CON group. While *f_Rikenellaceae*, *g_Rikenellaceae_RC9_gut_group* and *o__Peptostreptococcales-Tissierellales* were significantly enriched in FCSM group.

**Figure 5:**
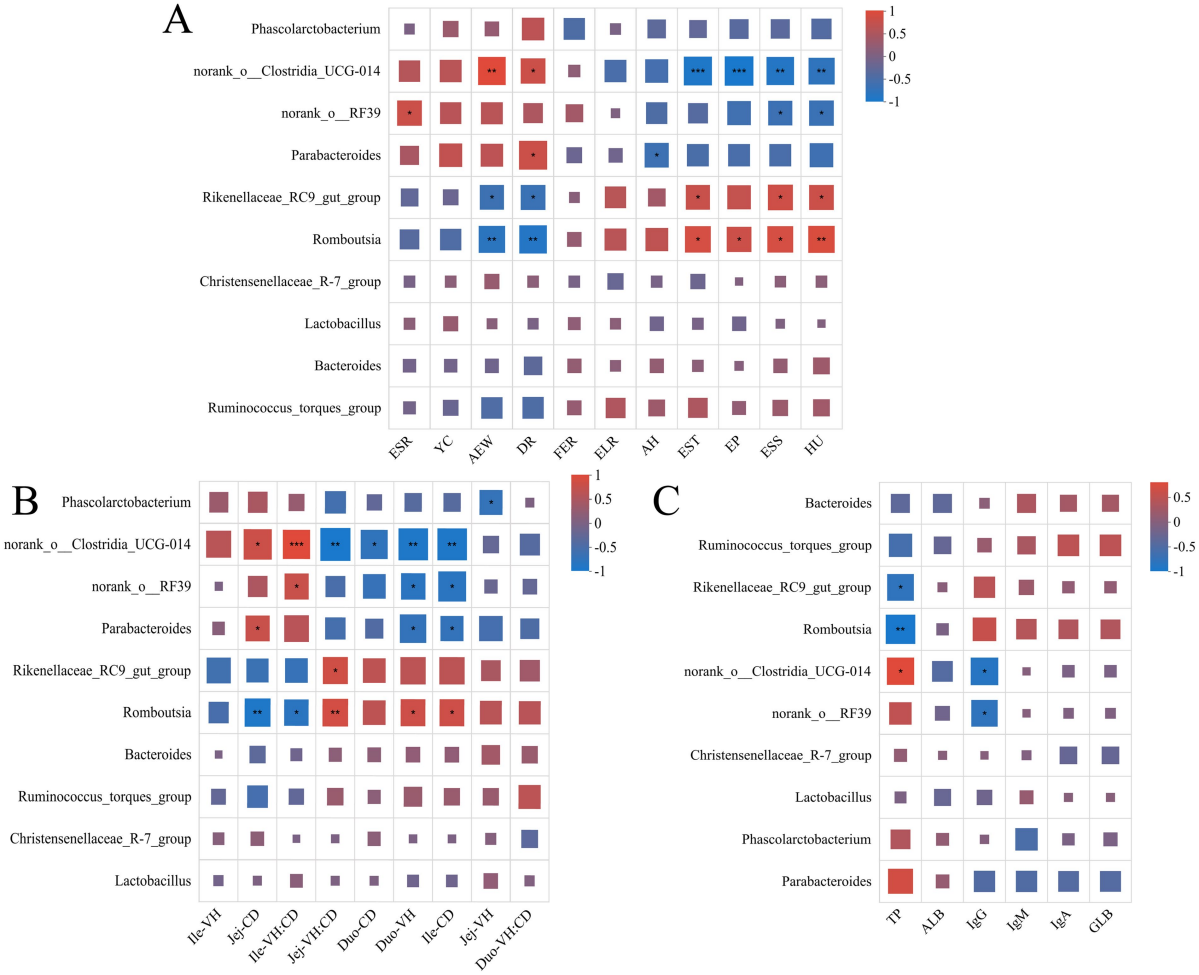
The heatmap of the relationship between environmental factors and intestinal flora. **(A)** Heat map of the correlation between the abundance and egg quality of the top ten genera. **(B)** Heat map of correlation with intestinal morphology. **(C)** Heat map of correlation with immune indicators. The X-axis and Y-axis are environmental factors and species, respectively, and the correlation R-value and *p*-value were obtained by calculation. R-values are represented by different colors in the graph, and the legend on the right side shows the color ranges of different R-values. “***” indicates *p* < 0.001. “**” indicates *p* < 0.01. “*” indicates *p* < 0.05.

In this study, we systematically investigated the association between intestinal flora and egg quality, immune indexes and intestinal morphology of laying hens under different feed systems by Spearman correlation analysis. The results showed that *Rikenellaceae_RC9_gut_group*, *Romboutsia* were significantly and positively correlated with egg quality parameters, such as Eggshell thickness, egg weight, Eggshell strength, Haugh unit, while they were significantly and positively correlated with Average egg weight and Death rate during egg laying period expression were negatively correlated. On the contrary, *norank_o_Clostridia_UCG-014*, *norank_o_RF39* and *Parabacteroides* were negatively correlated with egg quality parameters, but positively associated with Average egg weight, Death rate during egg laying period (Figure 5A).

In terms of gut morphology (Figure 5B), *Rikenellaceae_RC9_gut_group* and *Romboutsia* were positively correlated with jejunum VH: CD (Jej-VH: CD), duodenum VH (Duo-VH) and ileum CD (Ile-CD), while they were positively correlated with jejunum CD (Jej -CD) and ileum VH: CD (Ile-VH: CD). In terms of immune function, *Rikenellaceae_RC9_gut_group* and *Romboutsia* were negatively correlated with TP but positively correlated with other immune markers (e.g., IgG) (Figure 5C).

### 3.5 Metabolic analysis of FCSM

**Figure 6:**
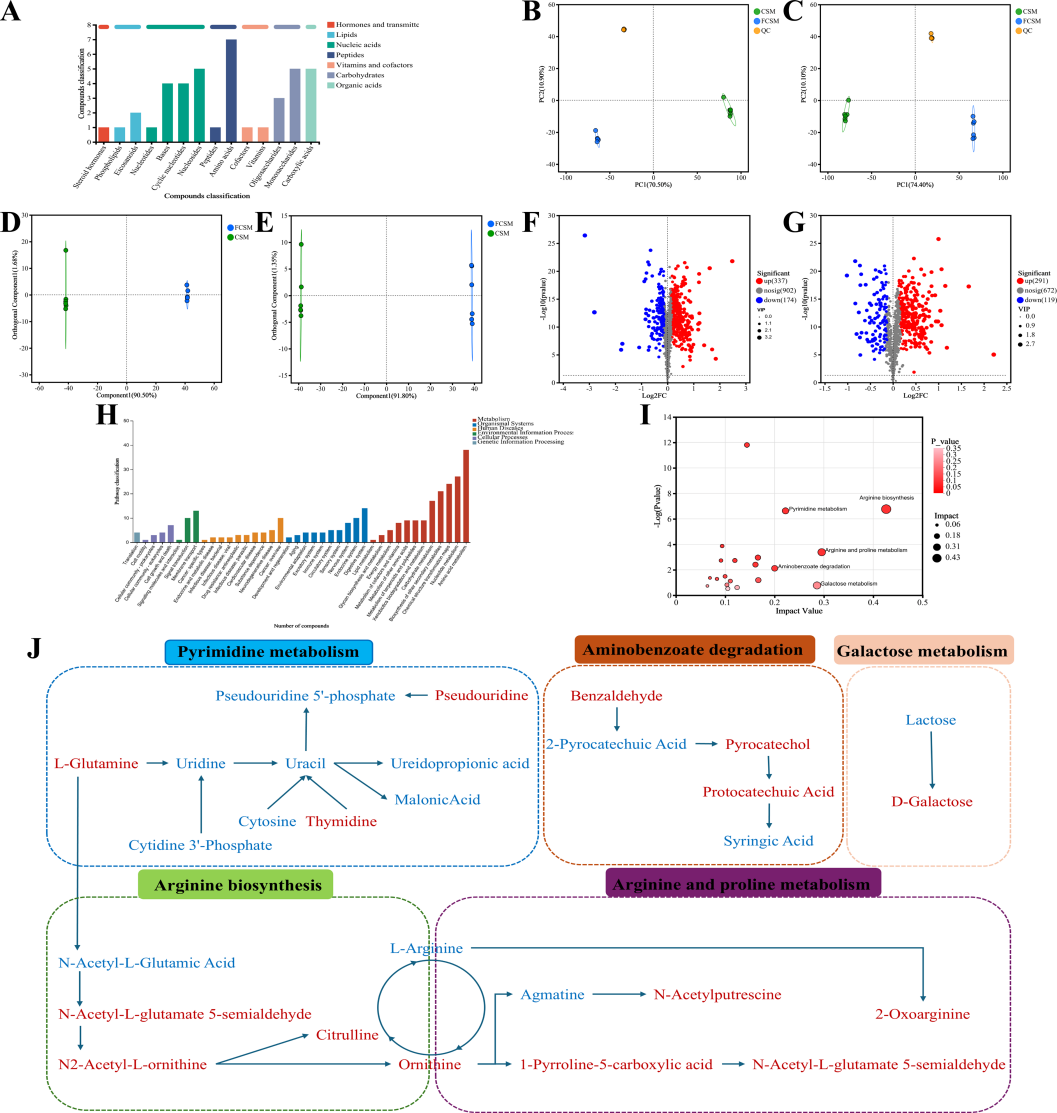
Untargeted metabolomics analysis of FCSM and CSM. **(A)** KEGG compound classification. **(B,C)** Principal component analysis (PCA) diagram in positive ion mode and negative ion mode. **(D,E)** OPLS-DA diagram in positive ion mode and negative ion mode. **(F,G)** Volcano map diagram in positive ion mode and negative ion mode. **(H)** Classification of KEGG database. **(I)** Bubble diagram of KEGG topology analysis. **(J)** Metabolic pathway visualization diagram.

In this study, the metabolites in FCSM were mainly composed of amino acids, nucleosides and monosaccharides, carboxylic acids (Figure 6A). The principal component analysis (PCA) plots for the positive and negative ion modes were shown in Figures 6B,C, the overlap of the quality control (QC) sample points indicated that this data is reliable. In addition, the separation of sample points in the CON and FCSM groups suggested that some differences in metabolites were produced in the two groups.

The overall difference in metabolites between the two groups was visualised by orthogonal partial least squares discriminant analysis (OPLS-DA) plots, with the FCSM group having a certain distance from the CON group, suggesting an altered metabolic profile of FCSM (Figures 6D,E). The R2Y values of the differential metabolites were generally higher than the Q2 values in the positive and negative ion models, indicating that the models were reliable and not overfitted (Supplementary Figures S2, S3). The volcano plot of differential metabolites showed that 337 metabolites were significantly up-regulated, and 174 metabolites were significantly down-regulated in the FCSM group in the cationic mode compared to the CSM. There were 291 metabolites significantly up-regulated and 119 metabolites significantly down-regulated in the anionic mode (Figures 6F,G). As a result, the key differential metabolites were distinguished and metabolite sets were created based on the KEGG database. There were 104 differential metabolites in the FCSM group compared to the CSM group. Therefore, we analyzed the trend of changes in differential metabolites in the CSM and FCSM groups by using a clustered heatmap. As shown in Supplementary Figure S4, Beneficial metabolites such as L-methionine, ornithine, allantoic acid, L-glutamine and citrulline, D-galactose, and thymidine increased after fermentation. To clarify the metabolic pathways where the differential metabolites were located among the experimental groups, we performed KEGG pathway annotation and enrichment analysis. The results indicated that the differential metabolites co-annotated by KEGG pathway were mainly involved in amino acid metabolism and nucleotide metabolism (Figure 6H). These differential metabolites could enhance the nutritional value of FCSM through pathways such as arginine biosynthesis, arginine and proline metabolism, galactose metabolism and pyrimidine metabolism, aminobenzoate degradation (Figure 6I).

**Figure 7:**
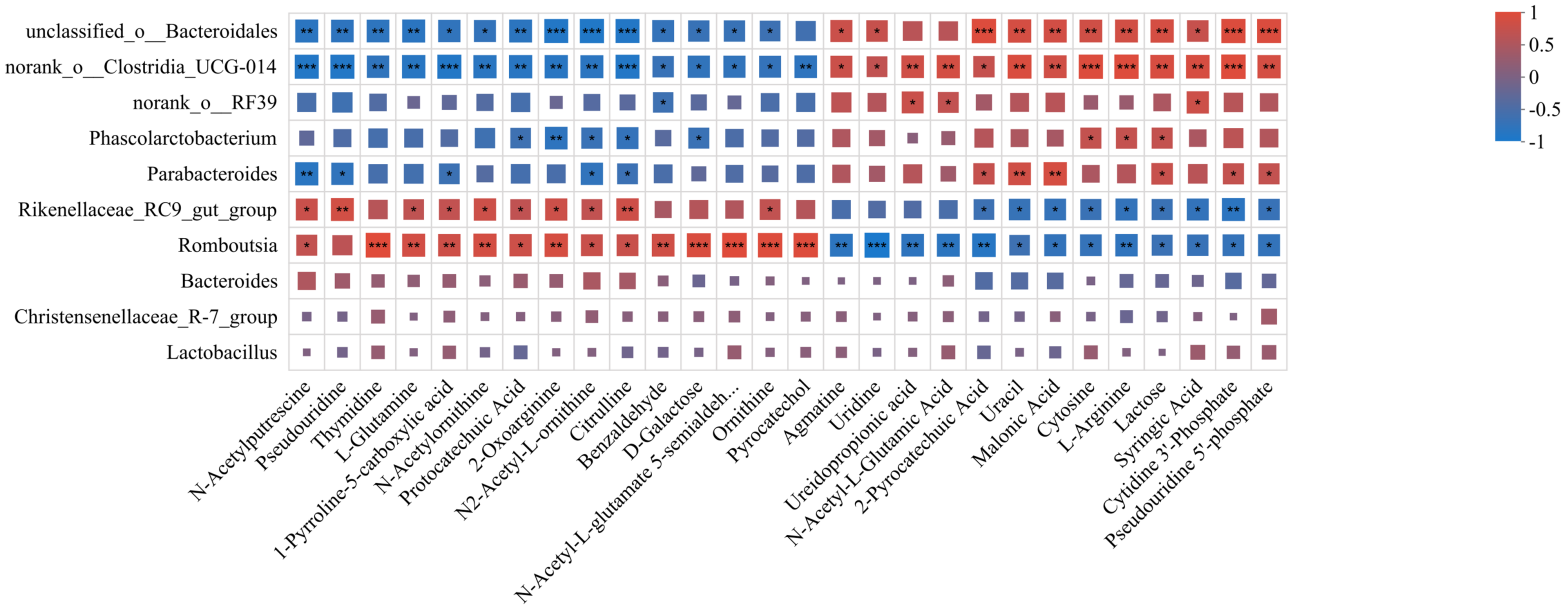
Correlation analysis of metabolites with intestinal flora. The X-axis and Y-axis are environmental factors and species, respectively, and the correlation R-value and *p*-value were obtained by calculation. R-values are represented by different colors in the graph, and the legend on the right side shows the color ranges of different R-values. “***” indicates *p* < 0.001. “**” indicates *p* < 0.01. “*” indicates *p* < 0.05.

The metabolic network of significantly different metabolites before and after fermentation was constructed (Figure 6J). The association between feed metabolites and gut flora was further explored by correlation analysis. The results showed that *Rikenellaceae_RC9_gut_group*, *Romboutsia* was positively correlated with ornithine, L-glutamine, citrulline and catechols, and negatively correlated with metabolites such as L-arginine, and lactose, among others. On the contrary, *norank_o_Clostridia_UCG-014*, *norank_o_RF39* and *Parabacteroides* were negatively correlated with ornithine, L-glutamine, citrulline and catechol, but positively correlated with metabolites such as L-arginine and lactose (Figure 7). These results suggested that the microbial fermentation technique may result in higher levels of nutrients in cottonseed meal, which in turn may improve the production performance and intestinal health status of laying hens fed FCSM-supplemented diets.

**Figure 8:**
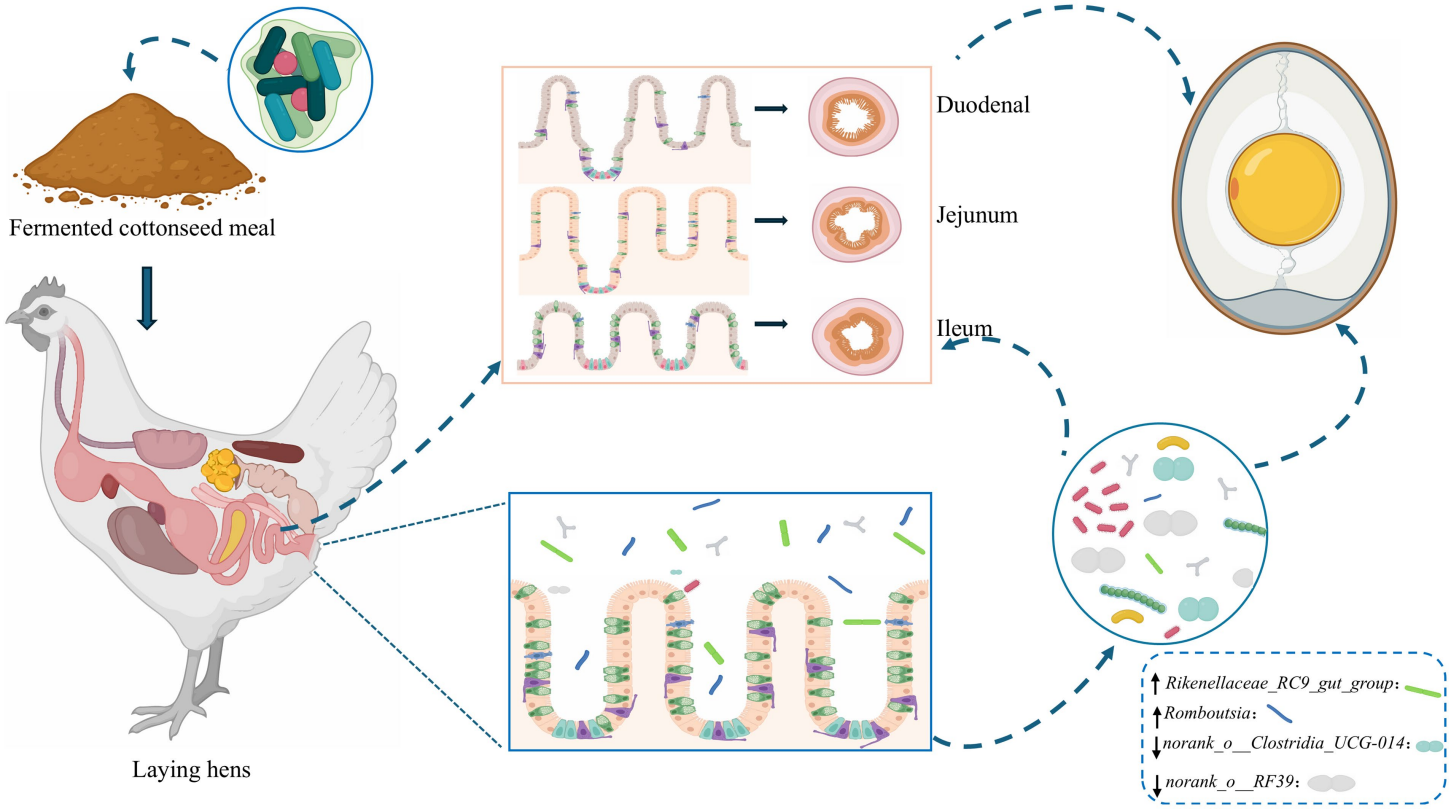
The sketch of the effects of FCSM on laying hens.

The main experimental contents and the sketch map were shown in Figure 8. Based on the above analysis, we concluded that the replacement of SBM by FCSM did not have adverse effects on laying hens, on the contrary, it also played a promotional role. In addition, we found that FCSM improved its nutritional value mainly through the action of the amino acid metabolism pathway.

## 4 Discussion

The toxic effect of FG on poultry presents systemic hazards (22). FG binds to proteins reduces nutrient absorption, and can cause edema and inflammation in organs such as the heart, liver, and kidneys as it circulates in the blood (23, 24). At the same time, FG causes anemia by chelating Fe^2+^, resulting in tissue hypoxia and metabolic disorders (25, 26). This is ultimately manifested by growth retardation, decreased production performance and lowered immunity. Monogastric animals, especially chickens, are sensitive to FG (27). In addition, the sensitivity of laying hens to FG is more significant due to reproductive vulnerability, risk of long-term exposure and differences in metabolic capacity (28, 29). Therefore, we further explored the effects of FCSM on the production performance and egg quality of laying hens. In this study, we found that FCSM suppressed the adverse effects of FG on laying hens, such as decreased production performance, reduced FER, yolk discoloration, and reduced egg quality and hatchability. Ashayerizadeh et al. (2) showed that using FCSM instead of SBM did not affect egg quality parameters and improved ESS and EST, which is consistent with the results of the present study. More importantly, the FER, EST, ESS, AH and HU of FCSM group were significantly better than those of CON group in this study. In addition, EST and HU of the FCSM group were significantly better than the international standard for quality eggs (EST ≥ 0.32 mm and HU ≥ 72). The organic acids and low pH in FCSM significantly promoted the dissolution and absorption of minerals such as calcium and phosphorus by lowering intestinal pH and improving intestinal structure and function (30, 31). A previous study found that increased serum calcium concentrations were positively correlated with improved eggshell strength in laying hens fed diets supplemented with FCSM (2). Therefore, the replacement of SBM by FCSM in diets may enhance the absorption and utilization of minerals in laying hens, ultimately improving eggshell quality, as well as reducing the cost of eggshell breakage and making eggs more suitable for the high-end market.

The thymus and bursa of laying hens gradually deteriorate at maturity during growth and development, so that their functional status cannot be assessed by organ indices (32). As the largest peripheral immune organ in laying hens, the relative weight of the spleen (organ index) is often used as an important indicator to assess the immune status of the body (33, 34). The developmental status of the spleen directly affects the ability of poultry to resist the invasion of pathogens, and is a key indicator for evaluating the growth performance and immune function of poultry (35). In this study, FCSM had no significant effect on the development and function of the spleen in laying hens. This result is consistent with existing studies and suggests that fermented feeds have potential advantages in maintaining poultry immunity or spleen health (36).

The immune function of animals is usually assessed by serum concentrations of serum proteins, immunoglobulins and cytokines, which play a key role in immunomodulation and disease resistance (37). Immunoglobulins (including IgG, IgM, IgA, IgE and IgD) are widely distributed in the blood, tissue fluids and exocrine secretions of poultry, of which IgG and IgM are the main effector molecules mediating humoral immunity, whereas IgA plays a central role in mucosal immunity, and together they are involved in the body’s defense mechanism (38). Long-term feeding of undetoxified CSM to laying hens, the FG in CSM absorbed by the intestinal tract, which can lead to lesions in the myocardium, hepatocytes, lungs, glomeruli, lymph nodes, etc., and ultimately cause damage to the immune organs and decrease in immunity or even trigger death. Therefore, FG residue is an important index to evaluate the safety of FCSM. In this study, FCSM did not impair the immune function of laying hens. The reduction of TP could be related to the efficient digestion and absorption of proteins in FCSM, which helped to optimize feed utilization and reduce feeding costs. This finding is consistent with the findings of Zhu et al. (39) that fermentation treatment could effectively reduce the antinutritional factors in CSM and improve its safety. In addition, FCSM promoted the effective utilization of nitrogen by regulating the structure of intestinal flora, thus reducing nitrogen emission and improving intestinal health (40).

As the main functional part of nutrient absorption, the morphology and structure of intestinal villi directly affect the absorption efficiency and utilization of nutrients (39). In the present study, FCSM was found to significantly increase the VH of duodenum in laying hens, which is similar to the findings of Lv et al. (26). This could be because carbohydrates in the FCSM were fermented by the intestinal flora to produce short-chain fatty acids (SCFAs). These SCFAs, especially butyric acid, not only activated the Wnt/*β*-catenin signaling pathway to promote the proliferation and differentiation of intestinal stem cells but also provided energy to the intestinal epithelial cells (40, 41). This in turn supported the growth and repair of intestinal epithelial cells and maintained the dynamic balance of villi and crypt structures (42). Additionally, the increase in intestinal VH significantly expanded the surface area for nutrient absorption, effectively promoting the efficiency of absorption of key nutrients such as amino acids, fatty acids, glucose, vitamins and minerals (43, 44). This provided more adequate nutrient substrates for follicle development and egg white protein synthesis in laying hens, thus supporting the process of high-quality egg formation at the molecular level (45–47). The reduction in the CD of jejunum may be related to the modulation of intestinal stem cell differentiation or the reduction of inflammatory response by FCSM (48). It has been shown that certain fermentation products may reduce intestinal inflammatory responses by inhibiting the NF-κB signaling pathway, thereby decreasing CD (49). On the other hand, changes in the VH/CD ratio reflected the different effects of FCSM on the function of different intestinal segments. The increase in the VH/CD ratio in the jejunum may be related to the enhancement of nutrient absorption, while the decrease in the VH/CD ratio in the ileum may be related to its function of regulating water and electrolyte absorption (50). Compared with the existing studies, the results of the present study were partially in agreement with those of Guo et al. (51) and Peng et al. (52), which indicated that fermented feeds could improve intestinal morphology and function in poultry. The present study further revealed the different effects of FCSM on the VH/CD ratios of jejunum and ileum, which provides a new perspective to investigate the mechanism of action of fermented feeds.

Being fed FCSM significantly affected the structure and function of the intestinal flora of laying hens. The relative abundance of Bacteroidetes was significantly increased by feeding FCSM. This may result from the fact that beneficial bacteria and metabolites produced by fermentation could provide favorable conditions for the growth of Bacteroidetes (53). Bacteroidetes are usually closely associated with host energy metabolism and immune regulation, and the increase in their relative abundance may reflect the positive regulatory effects of FCSM on the intestinal environment of laying hens (54–56). At the genus level, the relative abundance of *Rikenellaceae_RC9_gut_group* and *Romboutsia* increased significantly. Both genera can produce short-chain fatty acids (SCFAs). In addition, it has been shown that increased relative abundance of *Rikenellaceae_RC9_gut_group* may be positively correlated with immunomodulatory effects and improved metabolic functions in the host (57). As an important member of the intestinal commensal bacteria, *Romboutsia* exerts anti-inflammatory activity and antioxidant capacity through its metabolites, short-chain fatty acids, thus contributing to the maintenance of intestinal barrier integrity (58). Despite the significant increase in the relative abundance of *Romboutsia* in the present study, it has not been adequately reported in other similar studies. This may be related to the specific fermentation process of FCSM, such as strain selection and fermentation time. In conclusion, it was shown that FCSM could optimize the intestinal flora composition and improve intestinal health in laying hens. In the future, the interactions between different microbial groups and their effects on intestinal health and nutrient absorption in laying hens need to be explored in depth using multi-omics techniques.

In the present study, correlation analysis revealed the intrinsic mechanism of gut microbiota with production performance and health status of laying hens. The results showed that the relative abundance of *Rikenellaceae_RC9_gut_group* and *Romboutsia* was positively correlated with egg quality parameters (EST, ESS, HU). In contrast, the relative abundance of *norank_Clostridia_UCG-014*, *norank__RF39* and *Parabacteroides* was negatively correlated with egg quality, but positively correlated with AEW and DR. The *norank_Clostridia_UCG-014* and *norank_RF39* could affect egg quality by disrupting the intestinal barrier through the production of harmful metabolites such as ammonia and hydrogen sulfide, and by activating inflammatory pathways (59). *Parabacteroides* may exacerbate intestinal inflammation through the TLR4/NF-κB signaling pathway (60). Intestinal morphology correlation analysis showed that *Rikenellaceae_RC9_gut_group* and *Romboutsia* were positively correlated with VH: CD in the jejunum, VH in the duodenum and CD in the ileum. This may be because *Rikenellaceae_RC9_gut_group* and *Romboutsia* optimize nutrient absorption by promoting intestinal epithelial proliferation. On the contrary, *norank_Clostridia_UCG-014* and *norank_RF39* were positively correlated with CD in the jejunum and VH: CD in the ileum. This suggested that they may inhibit epithelial regeneration and lead to degradation of intestinal structure (61). Immunofunctional correlation analysis showed that *Rikenellaceae_RC9_gut_group* and *Romboutsia* were positively correlated with immune markers such as IgG, suggesting that they may enhance local immune responses through activation of gut-associated lymphoid tissues (62). The *norank_Clostridia_UCG-014* and *norank_RF39* were positively correlated with TP and negatively correlated with IgG, which may inhibit B cell differentiation and antibody production by inducing chronic inflammation (61).

Currently, microbial fermentation has been recognized as an effective method to improve the nutritional value of CSM. To deeply elucidate the nutritional value mechanism of FCSM, we used untargeted metabolomics to search for key metabolites. In this study, we discovered that FCSM group significantly increased nutritional value mainly through arginine biosynthesis, arginine and proline metabolism, and galactose metabolism. In another study, the solid-state fermentation of corn–soybean meal feed using compound strains was also mainly involved in arginine and proline metabolism (63). Excessive levels of arginine are present in CSM, which competitively inhibits lysine uptake (1). This imbalance would affect the efficiency of protein synthesis. In the present study, we found that fermentation resulted in a decrease in arginine levels in CSM, while increased levels of ornithine and citrulline were detected. It is hypothesised that arginine was converted to ornithine and citrulline during fermentation. This not only improved the amino acid imbalance but also provided raw material for protein synthesis in eggs. L-glutamine not only provided energy and metabolic substrates for intestinal epithelial cells to support normal cellular function but also enhanced the physical barrier function of the intestinal mucosa against bacterial and toxin attacks by supporting the synthesis of mucins (64). Zulkifli et al. found that dietary addition of L-glutamine increased intestinal villus length and crypt depth (65). L-glutamine, N2-acetyl-L-ornithine, and N-acetyl-L-glutamate-5-semialdehyde were interconnected in metabolic pathways that collectively affected production performance, antioxidant capacity, and intestinal health of laying hens by regulating nitrogen and energy metabolism. D-galactose as a prebiotic can regulate intestinal flora, enhance intestinal function, promote animal absorption and utilization of feed nutrients and enhance immunity. Spotted catfish experiments show that the addition of D-galactose fermented feed significantly regulates intestinal flora, optimizes carbohydrate absorption; interval feeding the fermented feed group showed stronger anti-inflammatory and immune ability (66). Catechol is an important intermediate in many organic synthesis reactions. It can be converted into a variety of useful compounds through oxidation, reduction, substitution and other reactions. In our previous study, we found that catechol is formed during the FG degradation pathway via catechol 23, dioxygenase, interstitial ring-opening, and enters the tricarboxylic acid cycle for complete degradation (67). In another study, catechol could also undergo interstitial cleavage by catechol 2,3-dioxygenase to produce 2-hydroxymuconate semialdehyde, which is further metabolized to pyruvate and acetaldehyde (68). Future absolute quantitative analyses of key metabolites in conjunction with targeted metabolomics approaches are needed to validate the results of untargeted metabolomics and to further complement and refine the metabolite composition. In addition, further studies are needed to explore the role of these key metabolites in regulating production performance and gut health in laying hens.

In this study, the core role of the interaction mechanism between the intestinal microbiota and the host in regulating the production performance of laying hens after feeding FCSM was systematically analyzed. In addition, and further explored the potential nutrients in FCSM to lay a theoretical basis for improving its nutritional value. Based on the present findings, future studies should focus on the precise interactions between colony-specific metabolites and the host to provide a scientific basis for the development of a new generation of functional feed additives.

## 5 Conclusion

In conclusion, the production performance and egg quality of laying hens in the FCSM group were better than those in the SBM group. In addition, this study found that FCSM could promote nutrient absorption and intestinal health by regulating intestinal flora, such as increasing the abundance of *Rikenellaceae_RC9_gut_group* and *Romboutsia*. Meanwhile, we also explored the dynamic changes of metabolites before and after CSM fermentation. Arginine and proline metabolism, galactose metabolism and pyrimidine metabolism, as well as aminobenzoate degradation, were identified as important metabolic pathways affecting the quality of FCSM. This not only broadens the application scope of unconventional protein resources but also lays a theoretical foundation for realizing the goal of sustainable development in the poultry industry.

## Data Availability

The original contributions presented in the study are publicly available. This data can be found here: GSA database (https://ngdc.cncb.ac.cn/gsa), with the accession number CRA028390.

## Footnotes

^1^ https://cloud.majorbio.com
