# Supplementary material for: Fermented cottonseed meal improves production performance, immune function and intestinal microecological environment of laying hens and its nutritional properties
Source: Front Vet Sci. 2025 Sep 22;12:1622229. doi: 10.3389/fvets.2025.1622229 (PMC12497634; doi:10.3389/fvets.2025.1622229)
Supplement: Supplementary file 1 [file Data_Sheet_1.docx]

Supplementary Material

Fermented cottonseed meal improves production performance, immune function and intestinal microecological environment of laying hens and its nutritional properties

Mingyang Xu^1^, Jia Li^1^, Wei Wang^1^, Tongguo Gao^1^, Caixuan Zhang^1,3^, Zhimin Hao ^1,^* and Chen Zhou^2,^*

^1^College of Life Sciences, Hebei Agricultural University, Baoding, Hebei 071000, China

^2^Central Laboratory, Affiliated Hospital, Hebei University, Baoding, Hebei 071000, China

^3^School of Light Industry Science and Engineering, Beijing Technology and Business University, Beijing 100000, China

*** Correspondence:**Zhimin Hao*
[hzm_0322@163.com](mailto:hzm_0322@163.com)

Chen Zhou*
[3154599585@qq.com](mailto:3154599585@qq.com)

Supplementary Table S1. Sensory evaluation of eggs.

| Items | CON | FCSM |
| --- | --- | --- |
| FO | 5.2±1.1^a^ | 5.1±1.3^a^ |
| AR | 7.2±0.9^a^ | 7.2±0.8^a^ |
| AF | 3.5±1.2^a^ | 3.2±1.1^a^ |
| OA | 7.1±0.7^b^ | 7.3±0.6^a^ |

Abbreviations: Fishy odor, FO; Aroma, AR; Aftertaste, AF; Acceptability, OA; control group, CON; experimental group, FCSM.


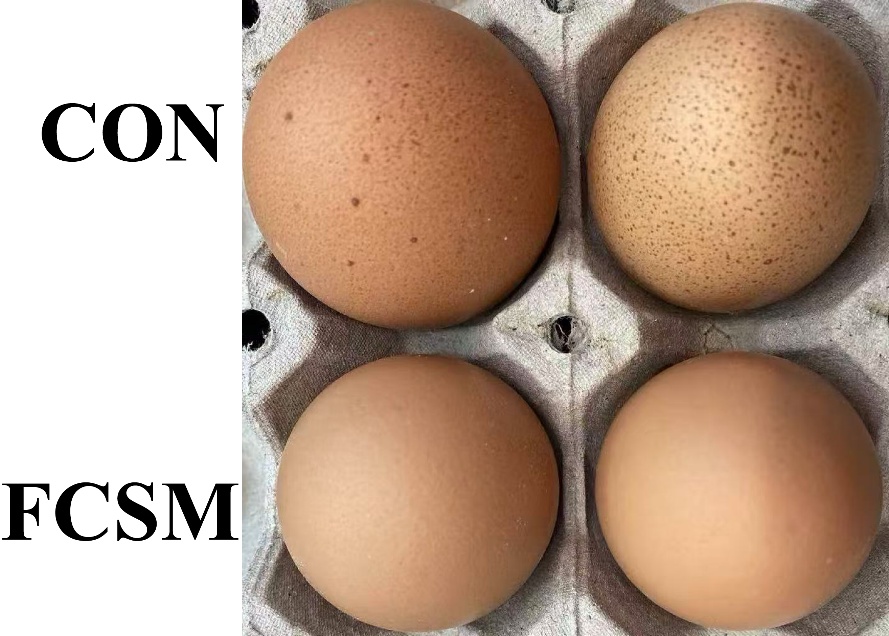


Supplementary Figure S1: Appearance and shape of eggs between CON and FCSM groups.


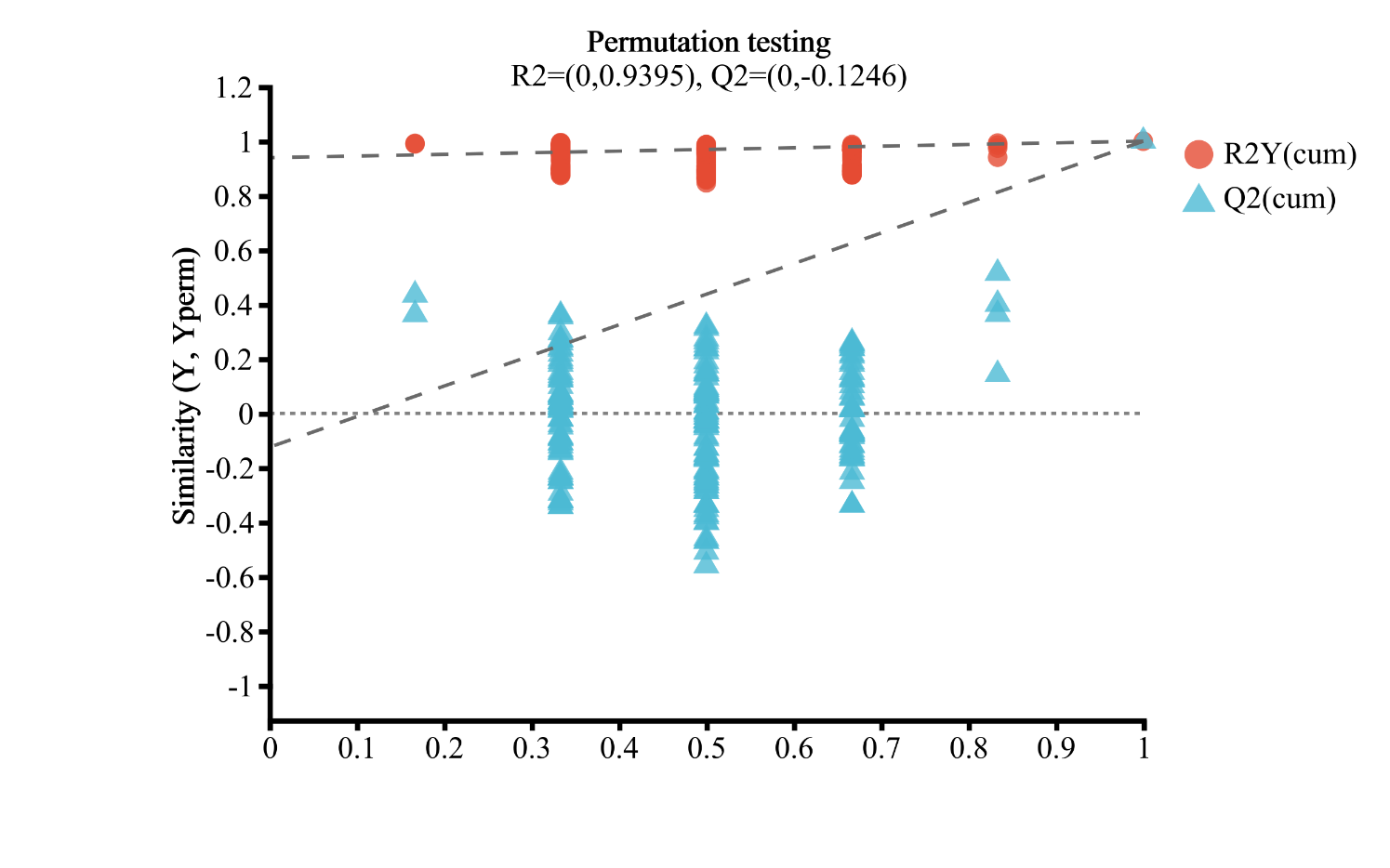
Supplementary Figure S2. OPLS-DA Displacement Test Chart Cation.


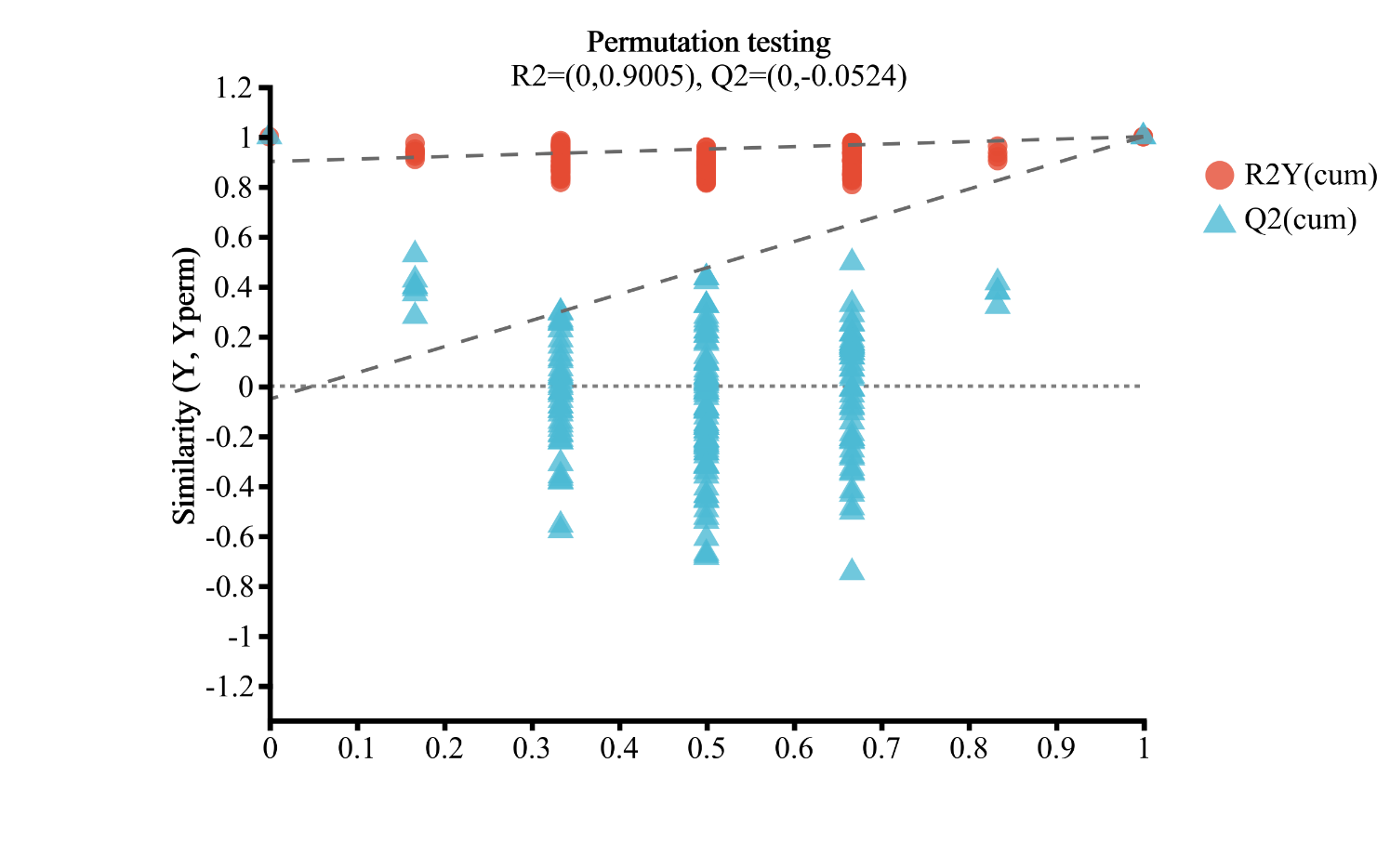
Supplementary Figure S3. OPLS-DA Displacement test plot anion.


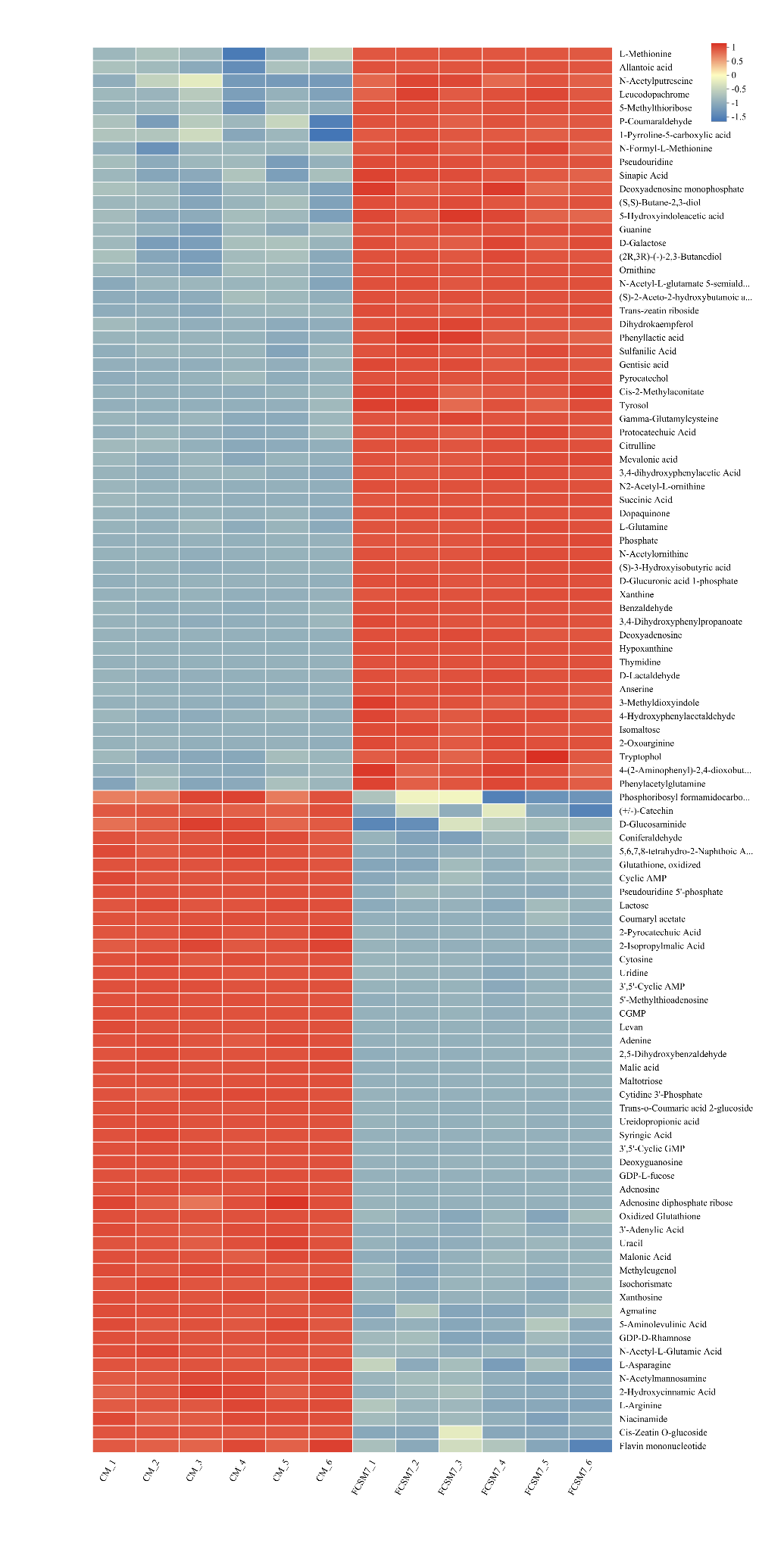


Supplementary Figure S4. Metabolite clustering analysis. Note: Each column in the figure represents one sample, each row represents one metabolite, and the color in the figure indicates the relative expression size of the metabolite in the group of samples. Please see the numerical notation under the color bar at the bottom right for the specific expression size change trend. The left side is the dendrogram of metabolite clustering, the right side is the name of metabolites, the closer the two metabolite branches are, the closer their expressions are; the upper side is the dendrogram of sample clustering, and the lower side is the name of the samples; the closer the two sample branches are, the closer the expression patterns of all metabolites in the two samples are, i.e., the closer the trend of the metabolite expression is.
